# Overprotective parenting and preschoolers’ physical activity and screen time: cross-sectional findings from the DAGIS survey

**DOI:** 10.1186/s12966-026-01910-3

**Published:** 2026-03-27

**Authors:** Jenna Rahkola, Henna Vepsäläinen, Reetta Lehto, Sanne Gerards, Jessica Gubbels, Josefine Kailaheimo-Björkqvist, Henna Launistola, Mirkka Maukonen, Nina Sajaniemi, Maijaliisa Erkkola, Eva Roos, Carola Ray

**Affiliations:** 1 Folkhälsan Research Center, Topeliuksenkatu 20, Helsinki, 00250 Finland; 2 Department of Food and Nutrition, University of Helsinki, P.O. Box 66, Helsinki, 00014 Finland; 3 Department of Health Promotion, Maastricht University, NUTRIM Institute of Nutrition and Translational Research in Metabolism, PO Box 616, Maastricht, 6200 MD The Netherlands; 4 Institute of Applied Health Sciences, University of Aberdeen, Foresterhill, Aberdeen, AB25 2ZD UK; 5 Finnish Institute for Health and Welfare, P.O. Box 30, Helsinki, 00271 Finland; 6 School of Applied Education Science and Teacher Education, University of Eastern Finland, P.O. Box 111, Joensuu, 80101 Finland; 7 Department of Public Health, University of Helsinki, PO BOX 20, Helsinki, 00014 Finland; 8 Department of food studies, Nutrition and Dietetics, Uppsala University, PO Box 560, Uppsala, 751 22 Sweden

**Keywords:** Physical activity, Active play, Screen use, Sedentary behavior, Digital media use, Parenting style, Helicopter parenting, parental control, early childhood

## Abstract

**Background:**

General parenting describes a parents’ overall approach to parenting across situations, creating the emotional climate of the parent–child relationship. Some general parenting constructs have been associated with children’s health behaviors. However, overprotective parenting—marked by excessive involvement and control with limited autonomy support relative to the child’s developmental level—remains largely understudied in relation to preschoolers’ physical activity (PA) and screen time (ST), as do the potential moderating effects of sociodemographic variables in these associations. Therefore, we examined the associations of overprotection with PA and ST among Finnish preschoolers and explored whether parental education or children’s sex moderated these associations.

**Methods:**

This cross-sectional study included 798 Finnish 3–6-year-olds and their parents from the DAGIS Survey. Parents completed an overprotection scale from an item-reduced version of the Comprehensive General Parenting Questionnaire. Children’s ST was parent-reported via a seven-day diary, while PA was measured with Actigraph accelerometers (for seven days). Weekday, weekend, and overall averages were computed for ST, moderate-to-vigorous physical activity (MVPA), and total physical activity (TPA). Linear mixed-effects models were used for analyses. The models were adjusted for the child’s age and sex, parental education, number of children in the household, birth order, and questionnaire respondent (mother/father). Moderation by sex and education was tested by adding the corresponding interaction terms to the models.

**Results:**

Children’s weighted averages were 71 min of MVPA, 398 min of TPA, and 76 min of ST per day. Overprotection was inversely associated with weekend MVPA (B: -3.49, 95%CI: -6.50; -0.48), weekend TPA (B: -9.94, 95%CI: -16.56; -3.34), and overall average TPA (B: -5.70, 95%CI: -10.78; -0.64). No associations with ST were found, nor were any associations moderated by sex or parental education.

**Conclusions:**

These results suggest an association between higher overprotection and lower PA among preschool-aged children regardless of child sex and parental educational level. We hypothesize that overprotective parents may, for example, restrict children’s PA due to safety concerns. Although the mechanisms and causality of the associations found require further investigation, identifying and addressing overprotective parenting could be beneficial in PA-promoting interventions.

**Supplementary Information:**

The online version contains supplementary material available at 10.1186/s12966-026-01910-3.

## Background

Higher levels of physical activity (PA) among preschoolers (3–6-year-olds) have been associated with a myriad of positive health and developmental outcomes, including lower risk for obesity and improved motor and cognitive development [1, 2]. On the other hand, high screen time (ST) in preschool-aged children has been linked with several unfavorable outcomes, such as sleep problems, higher risk for obesity, and compromised psychosocial well-being and development [3, 4]. According to a recent pooled analysis of data from 33 countries, 42% of 3–4-year-olds met the World Health Organization ST guideline (≤ 60 min/d), and 49% met the PA guideline (≥ 180 min/d total physical activity (TPA), including ≥ 60 min/d moderate-to-vigorous physical activity (MVPA)) [5]. Preschool age is an important period for fostering healthy PA and ST habits. It is marked by rapid growth and development, and the health-related habits established during this time tend to track into later life [6, 7], shaping long-term well-being. To design effective early interventions, understanding the determinants of PA and ST in this age group is essential.

Parents play a vital role in influencing children’s health behaviors, including PA and ST, and especially parenting practices (specific acts parents employ when socializing their children) related to PA and ST have been widely studied [8–10]. However, studies on general parenting in relation to PA and ST are lacking, particularly among preschoolers [8–10]. General parenting refers to a trait-like overall approach to parenting across situations and is thought to create an emotional and relational climate for parent–child interaction [11, 12]. There are various operationalizations and assessment methods for general parenting [12, 13]. One common approach distinguishes four parenting styles based on the dimensions of parental warmth and control: authoritative (high warmth, high control), authoritarian (low warmth, high control), permissive (high warmth, low control), and neglectful (low warmth, low control) [14, 15]. Some studies have examined associations between these styles or their dimensions and preschoolers’ PA and ST, with somewhat mixed results [16–19]. However, this approach has been argued to cover only limited aspects of general parenting [12, 13]. A further limitation is that it does not make full use of available data, and groups formed using median split (high/low) can be viewed as arbitrary [12, 14]. One general parenting construct not covered by this typology is overprotection (also called helicopter parenting).

The definitions and operationalizations of overprotection vary to some extent, but the following characteristics are often included. Overprotective parents are highly involved with the child and highly controlling—they closely supervise and govern the child’s activities while allowing little autonomy for the child [20–22]. Quick to intervene in challenges the child faces, they often discourage independent problem-solving, likely driven by a desire to ensure the child’s safety and well-being [20, 21]. Importantly, the degree of these behaviors is excessive or inappropriate relative to the child’s developmental level [21–23]. Although overprotective parenting shares some elements with the classic four‑style typology of general parenting [14, 15], it is generally considered a separate construct distinguished by a certain type of control and low support for child autonomy, and it is generally considered maladaptive [21–23]. However, research on its associations with children’s health behaviors is particularly scant.

In terms of children’s ST, one previous study has examined the association between parental overprotection and ST in preschool-aged children: higher parental overprotection was associated with higher odds of “excessive screen exposure” (> 4 h/day) among Turkish 2–5-year-olds [24]. Additionally, among adolescents and emerging adults overprotective/helicopter parenting has been linked with higher screen use described as addictive or problematic in a few studies [25–27]. Conversely, the association between overprotection and PA among preschool-aged children has not been examined previously, to our knowledge. One study among 7–12-year-old US children [28] did not find an association between helicopter parenting and PA participation frequency. However, three other “hyper-parenting” styles (little emperor, tiger mom, and concerted cultivation), which also seem to have some overlapping aspects with overprotection had an inverse association with PA frequency in that study [28]. Additionally, overprotection was not associated with PA among Belgian children aged 6 to 12 years [29]. While not directly examining overprotection or children’s PA, some literature suggests a potential association between the two. Some research has found that overprotective parents can be less likely to allow their children to engage in risky play and have a lower tolerance for it [30–32]. Risky play refers to “thrilling and exciting forms of play that involve a risk of physical injury” [33]. It is plausible to think that decreased risky play could associate with lower PA levels [34]. Moreover, a recent review identified parental concerns related to outdoor play or PA as a significant negative correlate of children’s (3–12 years old) outdoor play [35].

Socio-ecological and related frameworks of children’s health behaviors recognize that factors across various environmental settings can interact with each other and with the intrapersonal factors of the child in predicting behavior [36, 37]. Consistent with this perspective, a recent umbrella review regarding correlates of PA among 0–5-year-olds highlighted the need for research on such interactions [38]. Children’s sex and parental educational level (PEL) have been associated with general parenting [23, 29, 39] and children’s PA/ST [9, 35, 38, 40] in some studies, and findings among older children suggest that the associations between general parenting and children’s PA and ST may differ depending on children’s sex or PEL [40, 41].

Taken together, evidence on the role of general parenting—particularly overprotective parenting—in preschool-aged children’s PA and ST is both scarce and inconclusive, and potential moderators have yet to be identified. Because overprotection differs depending on children’s age [21, 23], it is imperative to examine these associations across various age groups. Moreover, previous studies have not examined these associations separately for weekdays and weekend days, despite reported differences in children’s PA and ST patterns between the two [42], and the greater amount of time parents presumably spend with their preschool-attending children on weekends. Hence, the objective of the current study was to: (1) examine the association of overprotective parenting with preschool-aged children’s PA and ST in Finland, and (2) explore whether children’s sex or PEL moderated these associations. Associations were separately examined for weekday, weekend, and overall average PA and ST. The overall average was included to capture children’s habitual behavior across the full week, complementing the weekday and weekend analyses and providing a more complete picture of the associations.

## Methods

### Design and participants

Our study sample consisted of children and their legal guardians (hereafter referred to as parents) who participated in the DAGIS (Increased Health and Wellbeing in Children, Families and Educational Settings) Survey in Finland between autumn 2015 and spring 2016 [36]. The cross-sectional DAGIS Survey is a part of the larger DAGIS research project that focuses on investigating and improving children’s health behaviors and related factors, with a special focus on health inequalities [36]. A total of 66 early childhood education and care (ECEC) centers from eight municipalities located in Western and Southern Finland participated in the DAGIS Survey. Via these centers, 864 children aged 3 to 6 years and their parents participated (24% participation rate) in the study. Written informed consent was obtained from the parents. A more detailed description of the sample selection and data collection of the DAGIS Survey can be found elsewhere [43]. The University of Helsinki Ethical Review Board in the Humanities and Social and Behavioural Sciences deemed the DAGIS survey ethically acceptable in February 2015 (#6/2015).

### Overprotective parenting

Overprotective parenting was assessed using an overprotection scale from an item-reduced version of the Comprehensive General Parenting Questionnaire (CGPQ) for 1–4-year-olds [22, 44]. The scale of the item-reduced version includes the following five items: “Every free minute I have, I spend with my child”, “I always help my child with everything he/she does”, “When my child cannot find something, I stop what I am doing to find it before he/she gets too upset”, “I do not let my child get involved in activities or tasks where he/she might get hurt”, “I carefully plan my child’s day so that he/she has enough activities to keep him/her busy”. In this study, one parent of a child rated the items on a five-point Likert scale (1 = Strongly disagree… 5 = Strongly agree). If more than one child from a family participated, this scale was completed separately for each child. As the final overprotection variable, a mean for the five items was calculated for each participant with no missing values. It was treated as a continuous variable in the analyses, with a higher value indicating more overprotective parenting. The psychometric validity of the item-reduced version of the CGPQ has been evaluated with satisfactory results [44]. Comparable to values reported in other samples [22, 23, 29], the Cronbach’s alpha for the overprotection scale in the current sample was 0.58.

### Children’s physical activity

Children’s PA was measured with hip-worn ActiGraph wGT3X-BT accelerometers (Pensacola, FL, USA) over a period of seven consecutive days, 24 h a day, excluding water-based activities. Research assistants set the accelerometers on children’s hips at the ECEC centers, and they were collected from the ECEC centers after the data collection period. Parents and preschool personnel received printed instructions on accelerometer use. Parents also reported the non-wearing time of the accelerometers in a diary. The raw data collected using 30 Hz were processed with ActiLife v.6.13.3 (ActiGraph, Pensacola, FL, USA) to obtain vertical axis activity counts for determining PA intensity metrics. An epoch length of 15 s was used, and periods with 10 min or more of consecutive zeros were defined as non-wear time and were excluded from further analyses [45]. Additionally, we excluded the times between sleep onset and wake-up that were reported by the parent. For a day to be considered valid, the child had to have worn the accelerometer for at least 600 min during waking hours. Subsequently, Evenson cut-points [46] were applied to define the time spent in the different PA intensities. Using criteria of at least three valid weekdays and one weekend day, we calculated the weekday mean, weekend day mean, and overall average ((5*weekday mean + 2*weekend mean)/7) for the time spent in MVPA and TPA (minutes/day). As a result, we obtained six continuous outcome variables of PA.

### Children’s screen time

The time the children spent sedentary on screens outside preschool hours was assessed using a seven-day parent-reported diary. This diary was adapted from a previously developed and validated (against accelerometry) instrument assessing PA and sedentary time in 3–5-year-olds [47]. For the DAGIS survey, it was modified to distinguish between television watching and DVD/video viewing and to include tablet/smartphone use as an option. The modified diary demonstrated good test–retest reproducibility in the DAGIS survey (intraclass correlation coefficient (ICC) for overall average ST = 0.65) [48]. Parents received the diary along with instructions and an example page. They were asked to report how much time (hours and minutes) their child spent sedentary (sitting or staying still) watching TV, watching videos/DVDs, using a tablet/smartphone, and using a computer/ playing computer games. Only screen use outside preschool hours and lasting longer than 10 min at a time was to be reported. The 10‑minute minimum duration was applied to support accurate parental recall of device‑specific screen use and to reduce reporting burden. The time spent on the different screen activities was summed up to obtain total ST for each available day. To compute the weekday mean, weekend mean, and overall average ((5*weekday mean + 2*weekend mean)/7) for ST (minutes/day), data from at least three weekdays and one weekend day were required. The three resulting continuous variables were used as outcomes in this study.

### Sociodemographic factors

Parents reported the child’s sex and date of birth, from which age was calculated. Parents also reported their own and their spouse’s educational level, the number of children currently living in their household, and the birth order of the child (only child, first child, middle child, or youngest child). Additionally, the relation of the questionnaire respondent to the child (mother, stepmother, father, stepfather, or other caregiver) was asked. A three-category variable for PEL was used in the analyses (low=comprehensive, vocational or high school; middle=bachelor’s degree or equivalent; and high=master’s degree, licentiate, or doctorate), indicating the highest education level among the parents of a child. These sociodemographic variables were used as potential confounders in the analyses, based on what has been used in previous studies [24, 28, 29], and reported correlates of general parenting, PA, and ST [9, 23, 35, 49]. Additionally, children’s sex and PEL were the potential moderators explored in this study.

### Statistical analyses

The analytic sample of our study consisted of participants who had data concerning overprotective parenting (*N* = 798). Within the analytic sample, 57 participants had missing values in the PA variables, and 60 participants had missing values in the ST variables. All analyses were performed with statistical software R version 4.4.2 [50], and a significance level of α = 0.05 was applied for all analyses. In each analysis, all participants with complete data on the variables needed for that analysis were included. The data were checked for outliers (≥ three interquartile ranges above or below the quartiles), and a few were found and removed from the ST variables: two from weekday screen time, three from weekend ST, and four from overall average ST. Using the T-test, Mann–Whitney U test, or Chi-Square test, we examined differences between participants with and without missing data regarding the key variables (overprotection, PA, or ST). Descriptive statistics were reported as frequencies, means, and standard deviations.

Linear mixed-effects models (“lme4” package version 1.1–30) [51] were used to examine the associations of overprotective parenting with children’s PA and ST, accounting for the clustering of children within families/households (the analytic sample comprised 707 households, and 192 children from households with more than one participating child). Accordingly, all models included a random intercept for family. ICCs of ≥ 0.4 supported using this modelling approach. Additionally, models incorporating random intercepts for preschool and preschool group were tested, but these were removed from the final models, as their ICCs were ≤ 0.07. In models with PA as the outcome, assumptions for residual normality and homoscedasticity were met (visual inspection of plots). For ST variables, a square root transformation was applied due to deviations from residual normality.

Direct associations between overprotective parenting and children’s PA and ST were examined with three models: (1) crude, (2) adjusted for the child’s age and sex, and (3) additionally adjusted for PEL, questionnaire respondent’s relation to the child, the child’s birth order, and number of children in the household (= fully adjusted model). This adjustment approach allows for distinguishing the contribution of child‑level characteristics from that of broader family‑level sociodemographic factors and for observing whether the associations remain robust across different levels of adjustment. Additionally, to adjust for differences in accelerometer wear time, all models with PA outcomes included the wear time as a covariate. To test whether children’s sex or PEL moderated the associations between overprotection and children’s PA or ST, interaction terms were added in a separate step in the linear mixed-effects models. The moderation was examined in separate models for children’s sex and PEL.

## Results

### Study sample descriptives

Table 1 displays the descriptives for the study variables. The mean age of the children was 4.8 years (SD = 0.9), and 52% of the children were boys. The overall average for ST was 76 min/day, while being 71 min/day for MVPA, and 398 min/day for TPA. The percentage of participants meeting the guideline for MVPA (≥ 60 min/d) was 71% on weekdays, 57% on weekends, and 69% when considering the weighted overall average. Middle-level education was the most prevalent PEL (42%), and most of the families had more than one child (87%). In addition, most of the questionnaire respondents were mothers (87%). Descriptives for the study variables are also presented by children’s sex and PEL in Supplementary Material 1 (Tables S1 and S2). Tests comparing participants with missing and complete data revealed a few statistically significant differences (Supplementary Material 2: Table S3): Participants with missing ST data had lower PEL (*p* < 0.001). They also had lower PA on weekdays (MVPA: *p* = 0.002, TPA: *p* = 0.004) and lower overall average PA (MVPA: *p* = 0.002, TPA: *p* = 0.003).

**Table 1:** Study sample descriptives (the DAGIS survey 2015–2016). *N* = 798

Variable		*N* (%)	Mean (SD)
Child’s age (years)			4.8 (0.9)
Child’s sex			
	Girl	381 (48)	
	Boy	417 (52)	
Questionnaire respondent			
	Mother	700 (87)	
	Father	95 (12)	
Parental educational level^a^			
	Low	180 (22)	
	Middle	334 (42)	
	High	281 (35)	
Child’s birth order			
	Only child	102 (13)	
	Firstborn	202 (25)	
	Middle child	88 (11)	
	Youngest child	397 (50)	
Number of children in the household			2.3 (1.0)
Overprotective parenting^b^			2.7 (0.6)
Moderate-to-vigorous physical activity (min/day)			
	Weekday		73 (23)
	Weekend		67 (26)
	Overall average^c^		71 (22)
Total physical activity (min/day)			
	Weekday		400 (49)
	Weekend		393 (60)
	Overall average^c^		398 (47)
Screen time (min/day)			
	Weekday		63 (35)
	Weekend		109 (63)
	Overall average^c^		76 (38)

a) low=comprehensive, vocational, or high school; middle=bachelor’s degree or equivalent; and high=master’s degree, licentiate, or doctorate

b) Overprotection scale (5 items) from an item-reduced version of the Comprehensive General Parenting Questionnaire (44), score range 1–5, with higher values indicating more overprotective parenting

c) Weighted average: (5*weekday mean + 2*weekend mean)/7

### Overprotective parenting and children’s physical activity

Results from the analyses examining the direct associations between overprotection and children’s PA are displayed in Table 2. In terms of MVPA, an association between overprotection and weekend MVPA remained statistically significant after all adjustments: children’s MVPA was lower if parents reported higher levels of overprotection (B=-3.49, *p* = 0.030). As for weekday and overall average MVPA, the associations did not remain statistically significant after adjustments. Regarding TPA, after all adjustments, children’s weekend TPA (B=-9.94, *p* = 0.003) and overall average TPA (B=-5.70, *p* = 0.023) were lower if parents reported higher levels of overprotection. The association between overprotection and weekday TPA did not remain statistically significant in the fully adjusted model.

**Table 2:** Association of overprotective parenting with children’s physical activity in the DAGIS survey data (2015–2016)

Outcomes (min/day)	Crude model *N* = 745–750		Age- and sex-adjusted model *N* = 745–750		Fully adjusted model *N* = 724–729	
	B	95% CI	B	95% CI	B	95% CI
Weekday MVPA	-2.97	-5.58; -0.35 *	-1.78	-4.20; 0.64	-1.47	-4.05; 1.11
Weekend MVPA	-4.07	-7.02; -1.12 **	-3.50	-6.36; -0.65 *	-3.49	-6.50; -0.48 *
Overall average^a^ MVPA	-3.15	-5.62; -0.68 *	-2.20	-4.50; 0.09	-1.98	-4.42; 0.46
Weekday TPA	-6.71	-11.88; -1.55 *	-5.93	-10.93; -0.94 *	-4.24	-9.53; 1.04
Weekend TPA	-10.79	-17.14; -4.46 **	-11.45	-17.78; -5.16 **	-9.94	-16.56; -3.34 **
Overall average^a^ TPA	-7.69	-12.64; -2.76 **	-7.37	-12.19; -2.58 **	-5.70	-10.78; -0.64 *

*Abbreviations*: *MVPA* Moderate-to-vigorous physical activity, *TPA* Total physical activity

All models adjusted for accelerometer wear time

Fully adjusted model adjusted for the child’s age and sex, parental educational level, birth order, number of children in the household, and questionnaire respondent (mother/father)

a) Weighted average: (5*weekday mean + 2*weekend mean)/7

* *p* < 0.05

** *p* < 0.01

### Overprotective parenting and children’s screen time

Results from the analyses examining the direct associations between overprotection and children’s ST are displayed in Table 3. No statistically significant associations were found between overprotection and children’s ST—neither for weekday, weekend, nor overall average ST.

**Table 3:** Association of overprotective parenting with children’s screen time in the DAGIS survey data (2015–2016)

Outcomes (min/day)	Crude model *N* = 739–755		Age- and sex-adjusted model *N* = 739–755		Fully adjusted model *N* = 719–752	
	B	95% CI	B	95% CI	B	95% CI
Weekday screen time	0.19	-0.04; 0.43	0.23	-0.01; 0.47	0.16	-0.09; 0.41
Weekend screen time	0.07	-0.27; 0.40	0.12	-0.21; 0.45	0.11	-0.23; 0.46
Overall average^a^ screen time	0.16	-0.08; 0.39	0.20	-0.03; 0.44	0.14	-0.11; 0.38

Screen time variables were square root transformed

Fully adjusted model adjusted for the child’s age and sex, parental educational level, birth order, number of children in the household, and questionnaire respondent (mother/father)

a) Weighted average: (5*weekday mean + 2*weekend mean)/7

* *p* < 0.05

** *p* < 0.01

### Moderation by children’s sex and parental education

Children’s sex or PEL did not moderate any associations between overprotection and PA or overprotection and ST. Results for each examined interaction term can be found in Table 4.

**Table 4:** Exploring moderation by children’s sex and parental education in associations between overprotective parenting and children’s physical activity and screen time in the DAGIS survey data (2015–2016)

Interaction term	Weekday		Weekend		Overall average^a^	
	B	95% CI	B	95% CI	B	95% CI
MVPA as an outcome (*N* = 719–729)						
Overprotection x sex (boy)	2.78	-1.73; 7.29	-0.08	-5.48; 5.30	1.96	-2.34; 6.26
Overprotection x education (middle)	-0.79	-7.45; 5.87	2.84	-4.98; 10.65	0.23	-6.08; 6.55
Overprotection x education (high)	2.40	-4.30; 9.12	5.08	-2.77; 12.93	3.12	-3.24; 9.48
TPA as an outcome (*N* = 724–729)						
Overprotection x sex (boy)	4.01	-5.22; 13.25	-0.84	-12.32; 10.63	2.61	-6.18; 11.39
Overprotection x education (middle)	2.96	-10.67; 16.57	-1.75	-18.89; 15.37	1.52	-11.56; 14.59
Overprotection x education (high)	2.03	-11.70; 15.74	-3.74	-20.99; 13.48	0.18	-13.00; 13.35
ST as an outcome (*N* = 719–735)						
Overprotection x sex (boy)	0.07	-0.33; 0.46	-0.02	-0.49; 0.45	-0.03	-0.41; 0.34
Overprotection x education (middle)	-0.14	-0.80; 0.51	0.61	-0.33; 1.54	0.13	-0.51; 0.78
Overprotection x education (high)	-0.64	-1.29; 0.02	0.30	-0.64; 1.23	-0.25	-0.89; 0.39

*Abbreviations*: *MVPA* Moderate-to-vigorous physical activity, *TPA* Total physical activity, *ST* Screen time

Reference categories were sex = girl; parental education = low

Models were adjusted for child’s age, birth order, number of children in the household, questionnaire respondent (mother/father), and parental education when child’s sex was examined as the moderator or child’s sex when parental education was examined as the moderator

Models with physical activity outcomes were additionally adjusted for accelerometer wear time

Screen time variables were square root transformed

Moderation by children’s sex and parental education were tested in separate models

a) Weighted mean: (5*weekday mean + 2*weekend mean)/7

## Discussion

This study examined how overprotective parenting associated with Finnish preschool-aged children’s PA and ST and whether these associations were moderated by the child’s sex or PEL. The results revealed an inverse association between overprotection and children’s PA, specifically weekend MVPA and TPA, along with overall average TPA. No associations were found between overprotection and children’s ST, nor did child’s sex or PEL moderate any associations in our sample. We will primarily interpret these findings with overprotection as the exposure and children’s PA/ST as the outcomes, while acknowledging that causality cannot be inferred from this cross-sectional study. Indeed, such associations can be bidirectional [52].

To the best of our knowledge, this study was the first to examine the association between parental overprotection and PA among preschool-aged children. Studies on older children are also scarce, and the results have been mixed [28, 29, 53]. The numerous measures used to assess overprotection and PA complicate the comparison of study results. None of the previous studies used accelerometers to measure PA. One previous study assessed overprotection with the CGPQ and did not find an association with parent-reported PA among 6–12-year-olds [29]. Furthermore, preschoolers’ activity typically takes place under parental supervision, whereas older children often engage in PA more independently, particularly in outdoor settings [54]. Hence, overprotection could be speculated to potentially have greater influence on PA during the preschool years.

Our finding that overprotection was associated with weekend MVPA only, but with both weekend and overall average TPA may indicate that overprotection is more influential outside preschool hours and may differentially affect activity intensity. This also suggests that the weekend patterns may be sufficiently pronounced to influence children’s overall weekly activity levels. As all children in this sample attended preschool (as do over 70% of all preschool-aged children in Finland [55]), the absence of an association with weekday PA may be explained by PA taking place at preschool—not under the direct influence of parents. Future studies could examine associations with PA specifically outside preschool hours. On the other hand, early educators’ approaches and practices can also shape children’s PA during preschool hours and may interact with parental behaviors [56]. Future studies could therefore consider early educators’ practices alongside parental overprotection and examine their potential interaction in relation to children’s PA.

One hypothesis on how overprotective parenting may lead to lower PA is that overprotective parents may restrict their children’s PA due to concerns about safety or injuries. This is suggested by one of the items in the overprotection scale of the CGPQ (“I do not let my child get involved in activities or tasks where he/she might get hurt”). Supporting this, prior research in preschool- and school-aged children has linked overprotectiveness with a lower tolerance for children’s risky play [30–32] and identified parental safety concerns as negative correlates of children’ s risky play and outdoor play [34, 35]. Lower engagement in risky play can be expected to mean less PA [34], but the intensity of the activity—light, moderate, or vigorous—remains unknown. Another hypothesis is that psychological factors, such as a child’s self-confidence, could mediate the association between overprotection and children’s PA. Higher parental overprotection has been associated with higher anxiety already in preschool-aged children [57], and with lower self-efficacy and self-control among emerging adults [27], and these factors can influence PA levels [58].

We did not find associations between overprotective parenting and children’s ST. This is inconsistent with some previous studies where higher overprotection has been associated with higher ST or problematic screen use mainly among adolescents and emerging adults [25–27], but also in 2–5-year-olds [24]. However, among 6–12-year-olds in Belgium, overprotection measured with the CGPQ was not associated with ST either [29]. Again, differing measures are likely to contribute to the differing results. In the diary used in this study, parents were instructed to report only ST episodes lasting ≥ 10 min, to facilitate estimation and reduce respondent burden. This approach may, however, underestimate total ST if a child engages in multiple shorter episodes. Moreover, low variability in ST may have limited the ability to detect an association in this study. When comparing with a study where higher overprotection was associated with excessive ST (> 4 h) among Turkish preschoolers [24], a smaller proportion of children in our sample had over four hours of ST. The absence of an association with ST is somewhat surprising given that we found an inverse association between overprotection and PA. Although the null result may be due to sample limitations, it could be fruitful to examine the association of overprotection with other sedentary activities.

Children’s sex or PEL did not moderate the associations between overprotection and PA or ST in this study, which contrasts some previous studies among older children that have examined other general parenting constructs and found moderation effects by sex or parental education [40, 41]. However, we are not aware of studies that have examined these moderation effects with overprotection. Some studies have reported differences in general parenting according to child sex and parental socioeconomic status, including education [39, 59]. One study, which specifically examined overprotection, reported lower levels among parents with higher education [23]. In terms of children’s movement behaviors, boys have consistently been found to be more physically active than girls [38]. Findings on the associations of sex with children’s ST [4, 9], and of PEL with children’s PA [38] have been mixed: many studies report null associations, while others show positive or negative associations. These inconsistencies appear to depend partly on the specific PA or ST domain examined [4, 9, 38]. The association between PEL and children’s ST is also inconsistent, but statistically significant findings mostly show an inverse association [9]. Nevertheless, our results suggest that the associations between overprotection and PA or ST are similar regardless of children’s sex or PEL. This needs to be confirmed in future studies, as we believe our study sample may have been limited particularly in ST variability.

A key consideration when interpreting the results of this study is that the data were collected in 2015–2016. Preschoolers’ movement behaviors, particularly screen use, have likely evolved since then, and the COVID‑19 pandemic introduced additional temporary disruptions (e.g., [60]). However, recent Finnish data from 2023 among 4–6‑year‑olds (*N* = 2010) [42] suggests that overall levels of MVPA and ST may not have changed substantially, although differences in assessment methods limit direct comparison. In that study, average MVPA was 74 min/day, and 56% of children had < 1 h of ST on weekdays and 18% on weekends (46% and 18% in our sample, respectively). Nonetheless, the composition of screen use has shifted over the last decade from predominately scheduled TV to on-demand streaming and application-based use on mobile devices, with free video‑sharing platforms now embedded in young children’s routines (e.g., [61]). Replication with more recent data—particularly considering device type and content—would help clarify whether these digital changes influence associations with parenting. Similarly, we cannot fully rule out the possibility that changes in the types of PA children engage in may influence the associations between parental overprotection and children’s activity.

All in all, our results suggest that overprotective parenting may be an important parental factor to consider in interventions aiming to promote preschool-aged children’s PA. Moreover, the null results regarding moderation suggest that there is no need to focus/tailor the interventions according to children’s sex or families’ educational background. Nevertheless, the causality and clinical relevance of the association between overprotection and PA is unclear at present. In our study, a one-point decrease in the overprotection score was associated with a 3.5-minute increase in weekend MVPA, which corresponds to 5.8% of the daily recommended MVPA for preschoolers, and with a 9.9-minute increase in weekend TPA (5.5% of the daily recommendation). Of course, since children’s PA is influenced by a plethora of factors [38], a large effect size for a single predictor is unlikely. Even if overprotective parenting was a minor contributor, it could still help identify families that may need more support in fostering children’s PA. How overprotection could be considered in interventions requires further investigation. It is unclear whether an overprotective parenting style can be modified. However, there is some evidence on successful interventions that have targeted general parenting [62]. Investigating potential mediators in the association between overprotection and children’s PA—such as PA‑related parenting practices, including restricting activities due to safety concerns—represents an important next step for clarifying the mechanisms underlying this relationship. Potential long-term effects of overprotection on PA and ST should also be studied. Additionally, because the overall parenting style of a parent likely reflects a constellation of aspects drawn from various dimensions/constructs of general parenting, future research could employ approaches, such as cluster analysis, to capture how these dimensions co-occur to form broader parenting style profiles.

Our study has several strengths. We were able to assess the association of overprotection separately with weekday PA/ST and weekend PA/ST that has not been done in previous studies. Strengths also include the use of validated measures for overprotection and PA. Hip-/waist-worn ActiGraph devices are commonly used to objectively measure PA among preschool-aged children [63]. Furthermore, the age group is an important strength related to our sample, as it is an important window for instilling healthy habits and an age when children are highly responsive to the impacts of parenting [7].

There are also limitations to consider. Regarding measurement methods, the limitations include parent-reported ST, which is susceptible to misreporting, e.g., due to social desirability bias [64]. However, diary use reduces recall bias compared with retrospective methods [64]. Not assessing children’s screen use during preschool hours may also be considered a limitation. However, we expect this to have minimal impact on our findings, as parental overprotection is unlikely to influence screen use occurring within preschool settings. Additionally, the Cronbach’s alpha (measure of internal consistency) of the overprotection scale was not optimal, although it can be deemed as acceptable [65]. Because several instruments exist to assess overprotection, and the construct itself is multidimensional, future research would benefit from efforts to refine its conceptualization and to standardize its measurement. Moreover, our sample has limitations. The DAGIS survey had a relatively low participation rate (24%), and although the sample included families from diverse socioeconomic backgrounds, parental education was higher compared with the general population in Finland [43]. Additionally, the children in this sample were, on average, sufficiently physically active, and missing ST data were more common among those with lower PA and lower PEL. Considering these sample characteristics and known patterns of non-participation in epidemiological studies [66], the associations observed here are likely to underestimate the true associations and may not be generalizable to all preschool-aged children. One more limitation related to the sample is its over-representation of mothers, calling for more studies on fathers’ overprotection. It will be important to know whether maternal and paternal overprotection have differing influences on children’s PA and ST. One key limitation of this study is its cross-sectional design. Although the models were adjusted for key covariates identified in previous literature, residual confounding cannot be fully excluded. Cross‑sectional data also limit the ability to establish temporal ordering or assess potential bidirectionality in the observed associations. Therefore, longitudinal studies are needed, and experimental designs would be required to draw robust causal inferences. Nonetheless, plausible pathways through which overprotection may influence children’s PA exist (though they require further empirical exploration) and cross‑sectional analyses provide valuable early evidence for emerging associations and help identify patterns that warrant further investigation.

## Conclusions

This cross-sectional study suggests that higher parental overprotection is associated with lower PA in preschool-aged children, and that the association does not differ by child sex or parental education. No associations were observed with ST, highlighting the importance of examining PA and ST separately within the same sample. These findings should be replicated with more diverse samples, and future research is needed to clarify the causal pathways and mechanisms underlying the association between overprotection and PA. Nevertheless, considering parental overprotection as a potential barrier to preschoolers’ PA may be useful in designing family-based interventions and in conversations between health care professionals and parents.

## Supplementary Information

Supplementary Material 1.

Supplementary Material 2.

## Data Availability

Researchers interested in the data from this study may contact principal investigator Eva Roos, eva.roos@folkhalsan.fi.

## Abbreviations

PA: Physical activity
ST: Screen time
TPA: Total physical activity
MVPA: Moderate-to-vigorous physical activity
PEL: Parental educational level
DAGIS: Increased Health and Wellbeing in Children, Families and Educational Settings
ECEC: Early Childhood Education and Care
CGPQ: the Comprehensive General Parenting Questionnaire
ICC: Intraclass correlation coefficient
