# Supplementary material for: Overprotective parenting and preschoolers’ physical activity and screen time: cross-sectional findings from the DAGIS survey
Source: Int J Behav Nutr Phys Act. 2026 Mar 27;23:47. doi: 10.1186/s12966-026-01910-3 (PMC13154676; doi:10.1186/s12966-026-01910-3)
Supplement: Supplementary file 1 — Supplementary Material 1. [file 12966_2026_1910_MOESM1_ESM.docx]

| **Table S1**. Study sample descriptives by child’s sex (DAGIS Survey data from 2015–2016). N=798. | | | | | | |
| --- | --- | --- | --- | --- | --- | --- |
|  | | Girls (N=381) | | Boys (N=417) | |  |
| Variable | | N (%) | Mean (SD) | N (%) | Mean (SD) | P-value |
| Child’s age (years) | |  | 4.7 (0.9) |  | 4.8 (0.9) | 0.30 |
| Questionnaire respondent | |  |  |  |  | 1.00 |
|  | Mother | 334 (88) |  | 364 (87) |  |  |
|  | Father | 44 (12) |  | 49 (12) |  |  |
| Parental educational level^a^ | |  |  |  |  | 0.20 |
|  | Low | 89 (23) |  | 91 (22) |  |  |
|  | Middle | 168 (44) |  | 166 (40) |  |  |
|  | High | 122 (32) |  | 159 (38) |  |  |
| Child’s birth order | |  |  |  |  | 0.24 |
|  | Only child | 42 (11) |  | 60 (14) |  |  |
|  | Firstborn | 90 (24) |  | 111 (27) |  |  |
|  | Middle child | 44 (12) |  | 43 (10) |  |  |
|  | Youngest child | 201 (53) |  | 195 (47) |  |  |
| Number of children in the household | |  | 2.3 (1.0) |  | 2.3 (1.0) | 0.88 |
| Overprotective parenting^b^ | |  | 2.7 (0.6) |  | 2.7 (0.6) | 0.67 |
| Moderate-to-vigorous physical activity (min/day) | |  |  |  |  |  |
|  | Weekday |  | 66 (20) |  | 80 (24) | <0.001 |
|  | Weekend |  | 61 (23) |  | 73 (28) | <0.001 |
|  | Overall average |  | 64 (19) |  | 78 (23) | <0.001 |
| Total physical activity (min/day) | |  |  |  |  |  |
|  | Weekday |  | 387 (48) |  | 412 (48) | <0.001 |
|  | Weekend |  | 384 (57) |  | 401 (62) | <0.001 |
|  | Overall average |  | 387 (45) |  | 409 (45) | <0.001 |
| Screen time (min/day) | |  |  |  |  |  |
|  | Weekday |  | 63 (34) |  | 63 (35) | 0.86 |
|  | Weekend |  | 106 (63) |  | 112 (62) | 0.19 |
|  | Overall average |  | 75 (38) |  | 77 (38) | 0.50 |
| a) low=comprehensive, vocational, or high school; middle=bachelor’s degree or equivalent; and high=master’s degree, licentiate, or doctorate.  b) Overprotection scale (5 items) from an item-reduced version of the Comprehensive General Parenting Questionnaire, score range 1–5, with higher values indicating more overprotective parenting.  P‑values are from chi‑square tests (categorical) and t‑tests (continuous). | | | | | | |

| **Table S2**. Study sample descriptives by parental educational level^a^ (DAGIS Survey data from 2015–2016). N=798. | | | | | | | | | |
| --- | --- | --- | --- | --- | --- | --- | --- | --- | --- |
|  | | | Low (N=180) | | Middle (N=334) | | High (N=281) | |  |
| Variable | | | N (%) | Mean (SD) | N (%) | Mean (SD) | N (%) | Mean (SD) | P-value |
| Child’s age (years) | | |  | 4.8 (0.9) |  | 4.7 (0.9) |  | 4.8 (0.9) | 0.57 |
| Child’s sex | | |  |  |  |  |  |  | 0.20 |
|  | | Girl | 89 (49) |  | 168 (50) |  | 122 (43) |  |  |
|  | | Boy | 91 (51) |  | 166 (50) |  | 159 (57) |  |  |
| Questionnaire respondent | | |  |  |  |  |  |  | 0.02 |
|  | Mother | | 163 (91) |  | 297 (89) |  | 235 (84) |  |  |
|  | Father | | 15 (8) |  | 33 (10) |  | 45 (16) |  |  |
| Child’s birth order | | |  |  |  |  |  |  | 0.16 |
|  | Only child | | 30 (17) |  | 36 (11) |  | 35 (12) |  |  |
|  | Firstborn | | 41 (23) |  | 78 (23) |  | 82 (29) |  |  |
|  | Middle child | | 24 (13) |  | 38 (11) |  | 25 (9) |  |  |
|  | Youngest child | | 82 (46) |  | 176 (53) |  | 136 (48) |  |  |
| Number of children in the household | | |  | 2.3 (1.0) |  | 2.4 (1.0) |  | 2.3 (0.8) | 0.49 |
| Overprotective parenting^b^ | | |  | 3.0 (0.6)^c^ |  | 2.6 (0.6)^d^ |  | 2.7 (0.6)^d^ | <0.001 |
| Moderate-to-vigorous physical activity (min/day) | | |  |  |  |  |  |  |  |
|  | Weekday | |  | 73 (24) |  | 72 (24) |  | 74 (23) | 0.77 |
|  | Weekend | |  | 69 (27) |  | 68 (27) |  | 65 (25) | 0.21 |
|  | Overall average | |  | 72 (22) |  | 71 (23) |  | 71 (21) | 0.82 |
| Total physical activity (min/day) | | |  |  |  |  |  |  |  |
|  | Weekday | |  | 397 (46) |  | 401 (51) |  | 401 (49) | 0.69 |
|  | Weekend | |  | 392 (56) |  | 396 (62) |  | 389 (61) | 0.42 |
|  | Overall average | |  | 396 (42) |  | 399 (49) |  | 399 (46) | 0.73 |
| Screen time (min/day) | | |  |  |  |  |  |  |  |
|  | Weekday | |  | 67 (32)^c^ |  | 64 (36)^cd^ |  | 58 (34)^d^ | 0.01 |
|  | Weekend | |  | 118 (62)^c^ |  | 112 (66)^cd^ |  | 100 (59)^d^ | 0.01 |
|  | Overall average | |  | 82 (36)^c^ |  | 78 (39)^cd^ |  | 70 (36)^d^ | <0.01 |
| a) low=comprehensive, vocational, or high school; middle=bachelor’s degree or equivalent; and high=master’s degree, licentiate, or doctorate.  b) Overprotection scale (5 items) from an item-reduced version of the Comprehensive General Parenting Questionnaire, score range 1–5, with higher values indicating more overprotective parenting.  P‑values are from chi‑square tests (categorical) and t‑tests (continuous).  Different superscript letters indicate statistically significant differences between groups. | | | | | | | | | |
