# Supplementary material for: Overprotective parenting and preschoolers’ physical activity and screen time: cross-sectional findings from the DAGIS survey
Source: Int J Behav Nutr Phys Act. 2026 Mar 27;23:47. doi: 10.1186/s12966-026-01910-3 (PMC13154676; doi:10.1186/s12966-026-01910-3)
Supplement: Supplementary file 2 — Supplementary Material 2. [file 12966_2026_1910_MOESM2_ESM.docx]

| **Table S3**. Examining differences between participants with and without missing data on physical activity, screen time, and overprotection (DAGIS Survey data from 2015–2016). | | | | | | | | | |
| --- | --- | --- | --- | --- | --- | --- | --- | --- | --- |
|  | Physical activity | | | Screen time | | | Overprotection | | |
| Variable | Missing (mean/count) | Not missing  (mean/count) | P-value | Missing  (mean/count) | Not missing (mean/count) | P-value | Missing (mean/count) | Not missing (mean/count) | P-value |
| Child’s sex |  |  | 0.909 |  |  | 0.420 |  |  | 0.906 |
| Boy | 43 | 409 |  | 48 | 404 |  | 35 | 417 |  |
| Girl | 41 | 370 |  | 36 | 375 |  | 30 | 381 |  |
| Child’s age (years) | 4.81 | 4.77 | 0.647 | 4.62 | 4.79 | 0.092 | 4.86 | 4.76 | 0.365 |
| Weekday ST (min/day) | 67.78 | 62.79 | 0.581 |  |  |  | 69.61 | 62.78 | 0.785 |
| Weekend ST (min/day) | 108.75 | 108.93 | 0.784 |  |  |  | 109.05 | 108.91 | 0.587 |
| Overall average^a^ ST (min/day) | 82.16 | 75.99 | 0.575 |  |  |  | 81.67 | 76.08 | 0.964 |
| Weekday MVPA (min/day) |  |  |  | 63.43 | 73.98 | **0.002** | 78.52 | 73.03 | 0.188 |
| Weekend MVPA (min/day) |  |  |  | 64.87 | 67.55 | 0.528 | 69.93 | 67.23 | 0.466 |
| Overall average^a^ MVPA (min/day) |  |  |  | 63.35 | 72.25 | **0.002** | 76.27 | 71.44 | 0.193 |
| Weekday TPA (min/day) |  |  |  | 380.28 | 401.90 | **0.004** | 407.60 | 400.14 | 0.435 |
| Weekend TPA (min/day) |  |  |  | 380.21 | 394.19 | 0.118 | 405.30 | 392.60 | 0.123 |
| Overall average^a^ TPA (min/day) |  |  |  | 379.78 | 400.02 | **0.003** | 407.58 | 398.25 | 0.260 |
| Parental education^b^ |  |  | 0.160 |  |  | **<0.001** |  |  | 0.212 |
| Low | 25 | 175 |  | 37 | 163 |  | 20 | 180 |  |
| Middle | 27 | 328 |  | 26 | 329 |  | 21 | 334 |  |
| High | 31 | 273 |  | 21 | 283 |  | 23 | 281 |  |
| Number of children in the household | 2.42 | 2.33 | 0.754 | 2.50 | 2.33 | 0.423 | 2.55 | 2.34 | 0.366 |
| Overprotection score | 2.80 | 2.69 | 0.161 | 2.82 | 2.68 | 0.109 |  |  |  |
| Abbreviations: ST, screen time; MVPA, moderate-to-vigorous physical activity; TPA, total physical activity.  P-values from Chi-Square Test for categorical variables and from T-test or Mann-Whitney U Test for continuous variables, depending on the normality of the distribution.  a) weighted average: (5*weekday mean + 2*weekend mean)/7  b) low=comprehensive, vocational or high school; middle=bachelor’s degree or equivalent; and high=master’s degree, licentiate or doctor. | | | | | | | | | |
